# *Stenotrophomonas maltophilia* Infections in Pediatric Patients – Experience at a European Center for Pediatric Hematology and Oncology

**DOI:** 10.3389/fonc.2021.752037

**Published:** 2021-10-12

**Authors:** Stefan K. Zöllner, Stefanie Kampmeier, Neele J. Froböse, Heidrun Herbrüggen, Katja Masjosthusmann, Alijda van den Heuvel, Christian Reicherts, Andreas Ranft, Andreas H. Groll

**Affiliations:** ^1^ Infectious Disease Research Program, Center for Bone Marrow Transplantation and Department of Pediatric Hematology/Oncology, University Children’s Hospital Münster, Münster, Germany; ^2^ Intensive Care Medicine, Department of General Pediatrics, University Children’s Hospital Münster, Münster, Germany; ^3^ Pediatric Oncology & Hematology, Pediatrics III, University Hospital of Essen, Essen, Germany; ^4^ Institute of Hygiene, University Hospital Münster, Münster, Germany; ^5^ Institute of Medical Microbiology, University Hospital Münster, Münster, Germany; ^6^ Center for Bone Marrow Transplantation and Department of Medicine A, University Hospital Münster, Münster, Germany

**Keywords:** children, cancer, transplantation, *Stenotrophomonas maltophilia*, blood stream infection, pulmonary hemorrhage

## Abstract

*Stenotrophomonas maltophilia* is an important nosocomial pathogen in immunocom-promised individuals and characterized by intrinsic resistance to broad-spectrum antibacterial agents. Limited data exists on its clinical relevance in immunocompromised pediatric patients, particularly those with hematological or oncological disorders. In a retrospective single center cohort study in pediatric patients receiving care at a large european pediatric hematology and oncology department, ten cases of invasive *S.maltophilia* infections (blood stream infections (BSI), 4; BSI and pneumonia, 3, or soft tissue infection, 2; and pneumonia, 1) were identified between 2010 and 2020. Seven patients had lymphoblastic leukemia and/or were post allogeneic hematopoietic cell transplantation. Invasive *S.maltophilia* infections occurred in a setting of indwelling central venous catheters, granulocytopenia, defective mucocutaneous barriers, treatment with broad-spectrum antibacterial agents, and admission to the intensive care unit. Whole genome sequencing based typing revealed no genetic relationship among four individual *S.maltophilia* isolates. The case fatality rate and mortality at 100 days post diagnosis were 40 and 50%, respectively, and three patients died from pulmonary hemorrhage. Invasive *S.maltophilia* infections are an emerging cause of infectious morbidity in patients receiving care at departments of pediatric hematology and oncology and carry a high case fatality rate.

## Introduction

*Stenotrophomonas* maltophilia (formerly: *Pseudomonas* or *Xanthomonas maltophilia*) is an aerobic non-fermenting *Gram-*negative bacillus (NGNB) that can be found ubiquitously in the environment (1). Next to *Pseudomonas aeruginosa* and *Acinetobacter* spp., the organism is considered the third most frequent nosocomial pathogen among non-fermentative bacteria (2, 3).

Pneumonia and bloodstream infection (BSI) are the most common clinical manifestations of *S.maltophilia* infections. Less frequently, *S.maltophilia* can cause urinary tract infections, cholangitis, peritonitis, wound infections, eye infections, arthritis, meningitis, and endocarditis (4, 5). Patients with hematologic malignancies are at high risk for *S.maltophilia* infection because of chemotherapy-induced neutropenia and immunodeficiency. Frequent exposure to broad-spectrum antibiotics and the presence of central venous catheters further enhance the risk of *S.maltophilia* infection (6, 7). The rate of *S.maltophilia* BSI among BSIs in this patient population has been reported to be as high as 60% (8–11).

Treatment of *S.maltophilia* infection can be difficult because of the organisms inherent resistance to a variety of antibiotics (12, 13). Trimethoprim-sulfamethoxazol (TMP-SMX) is the drug of choice, and fluoroquinolones are the proposed alternative. Similar to the treatment of *Pneumocystis jirovecii* pneumonia, up to five-fold higher than regular doses of TMP-SMX are recommended for severe infections (5, 14). Thus, the therapeutic options for *S.maltophilia* infections are quite different from those available for other NGNB, and appropriate antimicrobial therapy is often delayed through ineffective treatment during initial empirical therapy (15). Accordingly, mortality rates are high in immunocompromised and critically ill patients (11, 16), with 30-day mortality rates of *S.maltophilia* BSIs ranging from 11% to 53% (8, 11–13, 17–19).

While series of adult cancer patients with invasive *S.maltophilia* infections have been published in regular intervals, few reports exist for pediatric patients with cancer and/or allogeneic hematopoietic cell transplantation (HCT) (20–22). We therefore analyzed the incidence, genetic relatedness, clinical course and outcomes of invasive *S.maltophilia* infections observed during the past ten years at our institution, a high volume European pediatric cancer center with an active allogeneic HCT program.

## Methods

### Study Design and Setting

The study was a retrospective observational single center cohort study of children and adolescents with oncological or hematological disease including patients with autologous or allogeneic HCT receiving care at the Department of Pediatric Hematology and Oncology of the University Children’s Hospital of Münster between January 2010 and July 2020 with the last follow-up in October 2020. The Department’s referral patterns and admission data at the time of the study have been reported recently (23). Patients with *S.maltophilia* infection or colonization were identified through the Hospital’s central electronic medical information system. Inclusion criteria were medical care at the Department of Pediatric Hematology and Oncology; a diagnosis of either solid tumor, hematological malignancy, a non-neoplastic hematological disorder, or status post allogeneic HCT; and microbiology confirmation of *S.maltophilia* in blood, usually sterile body sites or respiratory secretions in the presence of pneumonia. Patient demographics, disease related parameters, clinical course and outcome data were retrieved from the medical information system and analyzed. The primary endpoint of outcome was survival at day +100 post diagnosis. Written informed consent for data collection and analysis was obtained within the consent procedure for cancer treatment, HCT, and specialized medical care approved by the local institutional review board. Data collection was accomplished by a pseudonymized standardized case report form.

### Standard Operating Procedures

All patients received treatment for their underlying condition according to standard protocols of the German Society for Oncology and Hematology (GPOH) or individual recommendations of the respective study groups. Up to December 2014, antibacterial prophylaxis was given to patients undergoing HCT and consisted of penicillin, ciprofloxacin and metronidazole in allogeneic and penicillin and ciprofloxacin in autologous HCT recipients, respectively. Antibacterial prophylaxis was discontinued starting 2015. Initial empirical antibacterial therapy for fever and neutropenia consisted of ceftazidime plus gentamycin until December 2016 and was then replaced by piperacillin/tazobactam. Unstable patients were to start with meropenem plus vancomycin and were subsequently deescalated, as feasible. This regimen was also used for escalation in patients with fever persisting for more than 48-72 hours or a new fever after defervescence, with or without additional empirical antifungal therapy at the discretion of the attending physician. Suspected or proven infections were treated according to current management recommendations. All patients received TMP-SMX 8 mg/kg (max. 320 mg) twice weekly as prophylaxis for prevention of *Pneumocystis jirovecii* pneumonitis, and topical polyenes or azoles for prevention of oropharyngeal candidiasis. Prophylaxis with TMP-SMX was continued until three months after end of therapy in cancer patients and until immunoreconstitution in allogeneic HCT recipients. Standard antifungal prophylaxis consisted of fluconazole for allogenic HCT recipients, and either posaconazole or voriconazole for patients with acute myeloid leukemia or recurrent leukemia (24, 25). Blood cultures were drawn in case of fever and daily until defervescence and negative results. Aerobic and anaerobic cultures with age-appropriate blood volumes were obtained from each lumen of an indwelling catheter or from a peripheral vein, if no catheter was present. Respiratory cultures were obtained by tracheal aspiration in intubated patients (n=4) and by sputum induction in non-intubated patients, respectively. Cultures form other body sites were obtained only when infection was clinically or radiologically suspected or on a case-by-case basis to monitor bacterial colonization by swabs from the throat and the perianal region. All patients were routinely screened for colonization with methicillin-resistant *Staphylococcus aureus* by a combined swab from the throat and the nares at each hospital admission.

### Definitions

Blood stream infection was defined as ≥ one positive blood culture for either *S.maltophilia* or any other bacterial and fungal pathogens obtained in a patient with fever and other signs of infection, where present. Infections at other body sites were defined by clinical and/or radiographic criteria. Pulmonary infection was considered to be radiological evidence of pneumonic infiltrates together with detection of *S.maltophilia* in respiratory secretions and BSI. In the absence of documented BSI, respiratory evidence of *S.maltophilia* together with direct detection of *S.maltophilia* in the intraoperative tissue cultures, as in patient 3 after open abscess surgery (**Table 1**), was considered pulmonary infection.

Table 1Demographics, underlying condition and principal treatment, central venous cannulation, infection and colonization data, concomitant clinical data, treatment and outcome of ten pediatric patients with oncological or hematological disease including patients with autologous or allogeneic hematopoietic cell transplantation and invasive *S.maltophilia* infections.

**Table:** 

Patient No.	Gender	Age (years)	Diagnosis	Allo HCT; time after HCT (days)**	Chemotherapy	IST	CVC	SM BSI	SM Pneumonia	SM Tissue Infection	Concomitant SM Colonization
**1**	M	9.2	HLH	Yes (MMUD); 523	No	No	Yes	Yes	No	Yes ****	Skin
**2**	M	4.6	DSP Mutation*	No	No	No	Yes	Yes	No	No	Skin
**3**	F	11.6	ALL	No	Yes	No	Yes	No	Yes***	No	Anus, skin, trachea
**4**	F	15.8	VSAA	Yes (MRD); 63	No	Yes	Yes	Yes	Yes***	No	No
**5**	M	17.9	ALL	Yes (MUD); 231	No	Yes	Yes	Yes	Yes***	No	Anus, trachea
**6**	M	5.2	ALL	No	Yes	No	Yes	Yes	No	No	Skin, pharynx
**7**	M	14.7	ALL	Yes (MMUD); 15	No	Yes	Yes	Yes	No	No	Anus
**8**	F	11.8	ALL	Yes (MUD); 13	No	Yes	Yes	Yes	No	Yes ****	No
**9**	F	2.8	EwS	No	Yes	No	Yes	Yes	No	No	No
**10**	F	0.8	SCID	No	No	No	Yes	Yes	Yes	No	Anus, skin, pharynx

**Table:** 

Patient No.	Broad-spectrum Antibiotics	Defective Skin Barrier	CRP(mg/dL)	WBC(10³/µL)	ANC(10³/µL)	ICU Admission	Mechanical Ventilation	Concomitant BSI	CVC Removal	Antibiotic Treatment	Total Duration of Treatment (days)	Survival *****	Follow-up (days)
**1**	No	Yes	3.5	8.2	0.7	No	No	No	No	Meropenem; moxifloxacin	14	Yes	2053
**2**	Yes	Yes	11.3	27.5	21.7	Yes	Yes	Yes^1^	Yes	TMP-SMX; meropenem;moxifloxacin; tigecyclin	20	Yes	395
**3**	Yes	No	25.5	0.2	0	Yes	Yes	No	No	Meropenem; fosfomycin;tigecyclin	45	No	45
**4**	No	No	19.8	0	0	Yes	No	No	No	Meropenem; ciprofloxacin	1	No	2
**5**	Yes	Yes	37.9	0.3	0	Yes	Yes	Yes^2^	Yes	TMP-SMX; meropenem	2	No	3
**6**	Yes	No	6.1	0.7	0.2	No	No	No	Yes	Ceftazidim; colistin; moxifloxacin; tigecyclin	18	Yes	167
**7**	Yes	No	23.0	0	0	No	No	No	Yes	Ceftazidim; colistin; moxifloxacin; tigecyclin	27	Yes	497
**8**	Yes	Yes	3.3	0	0	Yes	No	No	Yes	Meropenem; colistin;moxifloxacin; tigecyclin	10	No	10
**9**	Yes	No	9.7	0	0	No	No	No	Yes	Meropenem; ciprofloxacin	15	Yes	2446
**10**	Yes	Yes	15.2	23.3	18.0	Yes	Yes	Yes^3^	Yes	Ceftazidim; tobramycin	14	No	79

ALL, acute lymphoblastic leukemia; ANC, absolute neutrophil count; BSI, blood stream infection; CRP, C-reactive protein; CVC, central venous catheter; DSP, desmoplakin; EwS, Ewing sarcoma; F, female; HCT, hematopoetic stem cell transplantation; HLH, hemophagocytic lymphohistiocytosis; ICU, intensive care unit; IST, immunosuppressive therapy; M, male; MMUD, mismatched unrelated donor; MRD, matched related donor; MUD, matched unrelated donor; SCID, severe combined immunodeficiency; SM, Stenotrophomonas maltophilia; WBC, white blood cell count; VSAA, very severe aplastic anemia.

* Associated with recurrent infections, especially skin; care at the Department of Hematology and Oncology; * Conditions in allo-HCT recipients: Patient 1, chronic graft-versus-host disease (GVHD) of the skin, off immunosuppression, low dose steroids (< 0.3 mg/kg prednisone equivalent); patient 4, primary graft failure; patient 5, chronic GVHD of the skin and the gastrointestinal tract, immunosuppression with sirolimus, anti-inflammatory antibodies, methylprednisolone 2 mg/kg/d; patients 7 and 8 were prior to engraftment. ** including pulmonary hemorrhage (please see **Figure 3** for details); * patient 1 had a catheter exit-site infection, and patient 8 had necrotizing fasciitis involving the lower extremities and buttocks. ** Four patients died in direct causal relationship to the infection from pulmonary hemorrhage (patients 3,4,5) and necrotizing fasciitis (patient 7) with multiorgan failure, and one patient (patient 10) died two months after completion of treatment from unrelated causes in hospice care.

^1^ Staphylococcus hemolyticus, Staphylococcus aureus, Enterococcus faecalis and Candida albicans in the week prior to diagnosis of S.maltophilia infection and another blood culture positive for Staphylococcus hemolyticus in the week after; ^2^ Escherichia coli, Enterococcus faecium, Staphylococcus epidermidis in the week prior to diagnosis of S.maltophilia infection; ^3^ Pseudomonas aeruginosa, Staphylococcus hemolyticus, and Enterococcus faecium in the week prior to diagnosis of S.maltophilia infection.

### Identification and Susceptibility Testing

Standard blood culture systems (BACTEC^®^, Becton Dickinson, Sparks, Maryland, USA) were used for detection of bloodstream isolates. Blood culture vials were incubated for up to 14 days. Subsequent species identification was performed by Matrix-Assisted Laser Desorption/Ionization Time of Flight-Mass Spectrometry (MALDI TOF MS^®^, Microflex, Bruker, Bremen, Germany). Susceptibility testing was done using disk diffusion method in accordance with the standards of European Committee on Antimicrobial Susceptibility Testing (EUCAST) and interpreted using zone diameter breakpoints (EUCAST clinical breakpoints [version 6.0]) for TMP-SMX.

### Whole Genome Sequencing-Based Typing

To determine the clonal relationship of *S.maltophilia* strains isolated from blood cultures, available isolates were subjected to whole genome sequencing (WGS)-based typing using the Illumina MiSeq platform (Illumina Inc., San Diego, USA) as described previously (26). Due to the retrospective character of this study, only four individual samples from four different patients were available for testing. Using SeqSphere+ software version 2.0 beta (Ridom GmbH, Münster, Germany), all coding regions were extracted and compared in a gene-by-gene approach (core genome multilocus sequence typing, cgMLST) using SM K279a strain (GenBank accession number AM743169.1) as a reference sequence. Instead of a published cgMLST scheme, which is not yet available, this *ad hoc* scheme was used to differentiate the cluster. SeqSphere+ software was used to display the clonal relationship in a minimum spanning tree. For backwards compatibility with classical molecular typing, i. e. MLST, the MLST sequence types were extracted from the WGS data *in silico*.

### Statistical Analysis

Statistical analyses were carried out with SPSS Statistics 26 (IBM Corporation, Armonk, NY, USA) software package. Overall survival (OS) was calculated from primary diagnosis to death or last follow-up. Comparison of the frequency of *S.maltophilia* infections over time and statistical exploration of associations between patient- and disease related parameters and mortality were performed by the Fisher’s Exact test; univariate and multivariate analyses were not performed due to the limited sample size. The level of statistical significance was set at p<0.05 (two-sided).

## Results

### Demographic and Clinical Characteristics

**Figure 1:**
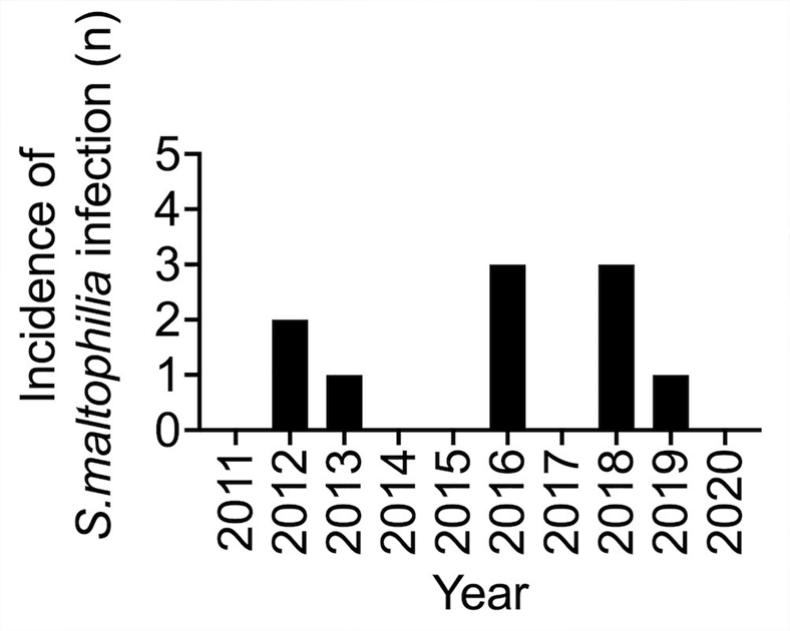
Annual frequency of *S. maltophilia* infection in children with oncological or hematological disease including patients with autologous or allogeneic hematopoietic cell transplantation between 2011 and 2020. For 2020, cases until March are included. p = 0.34 for the comparison of the proportion of *S. maltophilia* blood strem infections (BSI) (n = 9) among all BSIs in 2016-2020 relative to 2011-2015 (n.s.).

Between January 2010 and July 2020, a total of 502 distinct BSIs were identified in children with oncological or hematological disease including patients with autologous or allogeneic HCT receiving care at the Department of Pediatric Hematology and Oncology of the University Children’s Hospital of Münster. Of these, nine BSIs were due to *S.maltophilia*, accounting for a rate of *S.maltophilia* BSIs of 1.8% among all BSIs and of 7% among all *Gram*-negative BSIs, respectively. Considering one additional patient with documented pulmonary infection and positive cultures from all other sources, there were a total of ten invasive *S.maltophilia* infections in ten patients. Over time, there was a numerical, but not statistically significant increase in infected patients in the second half (2016–2020) of the study (**Figure 1**).

The demographic and clinical characteristics of the ten patients with invasive infections are listed in **Table 1**. Five patients each were male and female, and the median age was 10.4 years (range, 0.8 to 17.9 years). Five patients had acute lymphoblastic leukemia, and five patients had received allogeneic HCT and were between 13 and 523 days (median: 63) post-transplant. Seven patients were receiving antineoplastic or immunosuppressive therapy, and all had an indwelling central venous catheter at the time of diagnosis (Broviac-type, n=6; percutaneous transient catheter, n=3; port-a-cath-type, n=1). Among the 10 patients with invasive *S.maltophilia* infections, four had isolated BSIs, three a BSI and concomitant pneumonia, two a BSI and concomitant soft tissue infection, and one patient had pneumonia with an intrapulmonary abscess without positive blood cultures. In seven patients, superficial colonization by *S.maltophilia* was detected. Most affected patients (n=8) were receiving broad-spectrum antibacterial agents at the time of diagnosis, most frequently carbapenems (n=8), glycopeptides (n=7), and quinolones (n=6). All had an increased C-reactive protein level, and seven patients were profoundly granulocytopenic with an absolute neutrophil count < 500/uL. Six patients required admission to the intensive care unit at presentation, and four of these patients received mechanical ventilation because of pneumonia (n=3) and respiratory failure not related to pneumonia but to multiorgan failure (n=1) (**Table 1**).

### Concomitant Infections

Three patients were diagnosed with other BSIs in the week prior and/or the week after *S.maltophilia* infection and were receiving antibiotic treatment (patient 2 with *Staphylococcus hemolyticus, Staphylococcus aureus, Enterococcus faecalis* and *Candida albi*cans in the week prior and another blood culture positive for *Staphylococcus hemolyticus* in the week after; patient 5 with *Escherichia coli, Enterococcus faecium, Staphylococcus epidermidis* in the week prior; and patient 10 with *Pseudomonas aeruginosa, Staphylococcus hemolyticus*, and *Enterococcus faecium* in the week prior to *S.maltophilia* infection, respectively). Two patients (patient 4 and patient 5) showed concomitant low-level systemic *Epstein-Barr virus* reactivation, and one patient (patient 4) had systemic *Herpes simplex virus 1* reactivation (**Table 1**).

### Antimicrobial Susceptibilities and Genotyping

**Figure 2:**
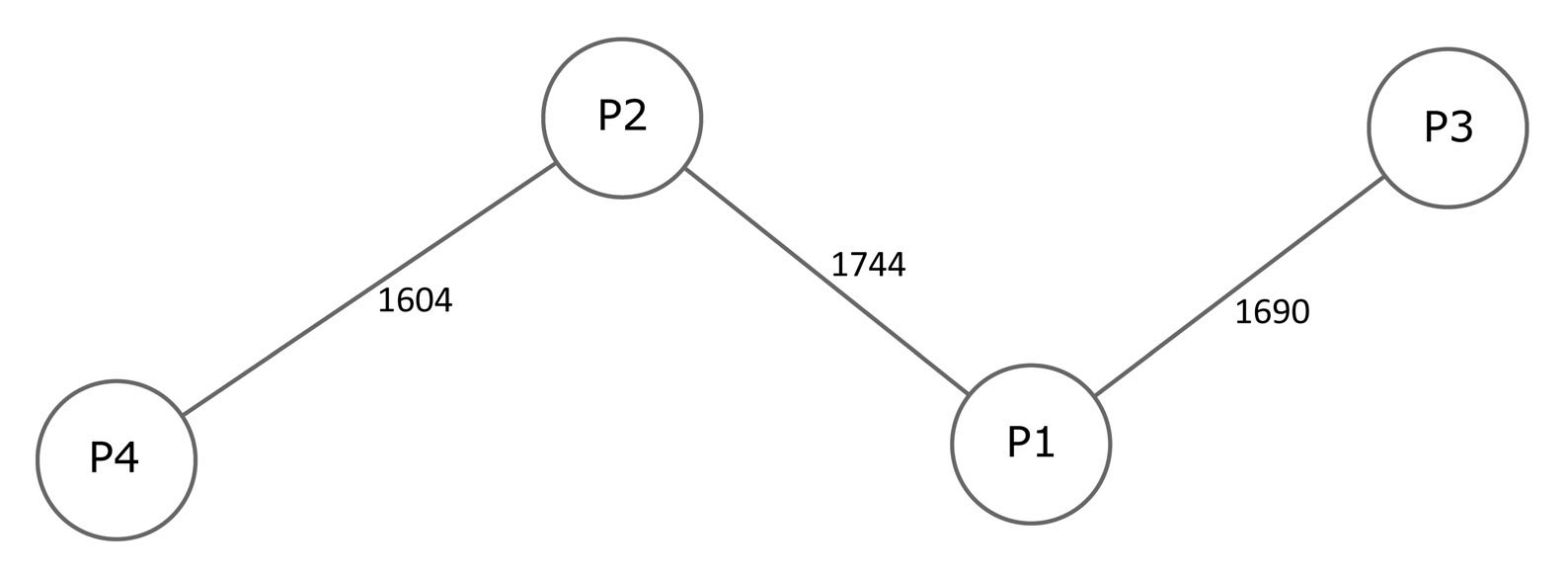
Minimum spanning tree of four *S.maltophilia* blood culture isolates obtained from four different patients. The tree is based on up to 1876 target genes, pairwise ignoring missing values. Each dot represents one genotype (P1-P4). Different connecting lines and numbers on these lines show the number of alleles differing between two genotypes.

Using disk diffusion methodology in accordance with the standards of European Committee on Antimicrobial Susceptibility Testing (EUCAST) and an agar diffusion diameter of > 16 mm assumed as susceptible (increased exposure), 70% of all ten initial isolates were susceptible to TMP-SMX. However, in one of the seven patients with a TMP-SMX-susceptible initial isolate, a follow-up blood stream isolate obtained three days after the initial one was tested non-susceptible. WGS-based typing and gene by gene comparison of four initial *S.maltophilia* blood culture isolates obtained from four different patients showed allelic differences between strains of at least 1604 alleles, thereby excluding any genetic relatedness of subjected *S.maltophilia* isolates (**Figure 2**).

### Antimicrobial Management and Outcome

**Figure 3:**
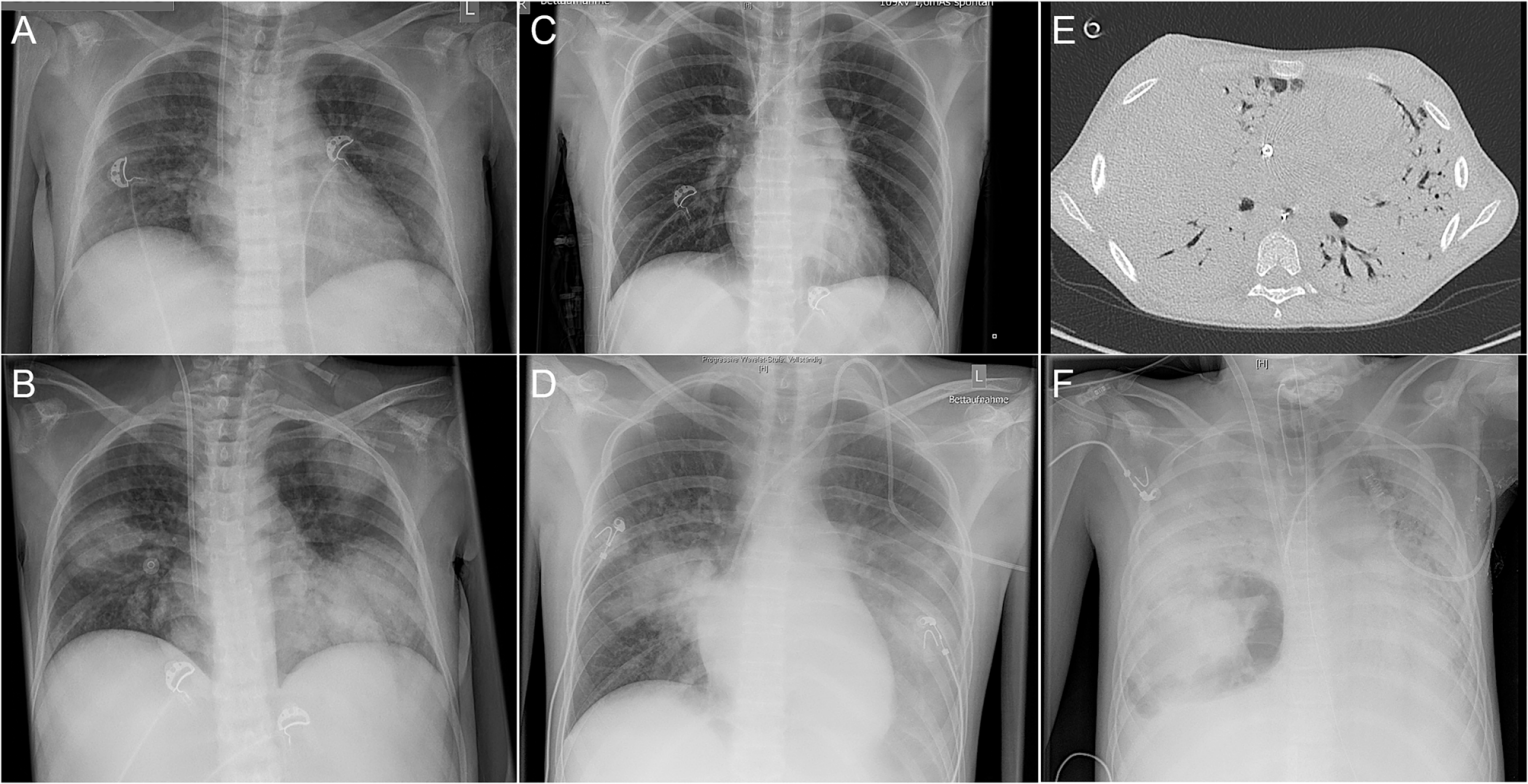
Radiographic findings in three patients with *S.maltophilia* infection and pulmonary hemorrhage. **(A, B)** 15-years old girl post allogeneic HCT for aplastic anemia (patient 4). **(A)** Normal chest x-ray at day +1 following allogeneic HCT; **(B)** *S.maltophilia*-related sepsis and ultimately fatal diffuse pulmonary hemorrhage at day +12 with detection of *S.maltophilia* in tracheal aspirates. **(C, D)** 17-years old male post allogeneic HCT for acute lymphoblastic leukemia (ALL) (patient 5). **(C)** Normal chest x-ray obtained during evaluation prior to transplantation; **(D)** *S.maltophilia*-related sepsis with ultimately fatal diffuse pulmonary hemorrhage on day +1 post-transplant. **(E, F)** 11-years old female with ALL (patient 3). First tracheal detection of *S.maltophilia* four days after microbiologically proven methicillin-susceptible *Staphylococcus aureus* pneumonia. **(E)** Middle lobe bleeding and atelectasis on chest CT-scans twenty-four days after first *S.maltophilia* detection. **(F)** Intrapulmonary abscess thirty-one days after first *S.maltophilia* detection; in the context of surgical resection, documentation of *S.maltophilia* from intraoperative tissue. Death forty-five days after first *S.maltophilia* detection.

The indwelling central venous catheter was removed shortly after diagnosis in seven of the nine patients with positive blood cultures. One patient (patient 7, **Table 1**) received repeated granulocyte transfusions. Antimicrobial treatment of *S.maltophilia* infection was highly heterogeneous and included combinations of meropenem (7), fluoroquinolones (7), tigecyclin (5), colistin (3), TMP-SMX (2), ceftazidime (1), fosfomycin (1), and tobramycin (1) administered for a total treatment duration of 1 to 45 days (median: 14.5 days). Of note, in retrospect, it is difficult to distinguish precisely between therapy directed at *S.maltophilia*, empiric treatment for suspected infections or directed treatment of confirmed concomitant infections, but the agents TMP-SMX and moxifloxacin were added only when *S.maltophilia* was detected. The 30-day mortality rate and the overall mortality rate were 30% and 50%, respectively, after a median follow-up time of 123 days (range, 2 to 2446 days). Four patients died in direct causal relationship to the infection after 2, 3, 10 and 45 days after diagnosis from pulmonary hemorrhage (patients 3,4,5, **Figure 3**) and necrotizing fasciitis (patient 7) with multiorgan failure (**Table 1**). Explorative statistical analysis of factors associated with overall mortality in patients with invasive *S.maltophilia* infections revealed the presence of pneumonia (p=0.047) and admission to the intensive care unit (p=0.047) as being associated with dismal outcome (**Supplementary Table 1**).

## Discussion

*Stenotrophomonas maltophilia* is a non-fermentative, *Gram-*negative bacillus that has emerged as important nosocomial pathogen in immunocompromised and critically ill patients (16, 27). Published experience in pediatric patients with cancer and/or allogeneic HCT is limited to two separate studies reporting on a total of 24 *S.maltophilia* BSIs (22, 28) and several larger pediatric series that include a relevant proportion of patients with hematological malignancies or solid tumors (24, 25, 29, 30) (**Table 2**). In the study presented here, *S.maltophilia* accounted for 1.8% of all BSIs and for 7% of those caused by *Gram-*negative rods. Invasive *S.maltophilia* infection was associated with a diagnosis of acute leukemia and/or allogeneic HCT, or immunodeficiency, and occurred in a setting of impaired host defences, defective mucocutaneous barriers, indwelling central venous catheters, treatment with broad-spectrum antibacterial agents, and admission to the intensive care unit. Four patients died in direct relationship to the infection, including three patients with pneumonia and pulmonary hemorrhage (**Figure 3**), which has been reported to be associated with *S.maltophilia* infection and status post allogeneic HCT (29, 44, 45). Similar to others (28), we found a numerical increase in *S. maltophilia* infections over time. Molecular typing of a limited number of blood culture isolates, however, confirmed that isolates were genetically not related and suggests the absence of a nosocomial outbreak (46).

**Table 2:** Literature overview of case series reporting blood stream infections of *S. maltophilia* in pediatric patients.

Patient Collective	Study Duration (years)	*S. maltophilia* Pts. (number)	Isolates (number)	Source	All-cause Crude Mortality (%)	Attributed Mortality (%)	Risk Factors (RF)	Positive Effect on Survival	Reference	Publication Date
**Pediatric pts**	6.5	79	85	non-respiratory	12.5	6.3	NA	NA	(31)	2000
**Pediatric pts**	5	8	8	blood	NA	NA	NA	NA	(32)	2002
**Pediatric** **cancer pts**	4	6	6	blood	NA	NA	NA	NA	(28)	2006
**Infants <180 days** **with heart disease**	5	32	47	blood, CSF, urine, eye, wound, BAL	37.5	NA	RF for outcome: prolonged positive SM cultures (p=0.008) need for renal dialysis (p=0.04) presence of stroke (p=0.05)	outcome-related: High ALC prior infection (p=0.01) Less mechanical ventilation days (p=0.006)	(33)	2015
**Pediatric pts**	5	18	18	blood	NA	NA	NA	NA	(34)	2016
**Pediatric pts**	2	19	NA	blood	NA	NA	RF for BSI: prior use of carbapenems within 7 d (p=0.02) prior ICU stay (p=0.03) mechanical ventilation (p=0.01)	BSI-related: Consultation with ID physician (p=0.04)	(35)	2016
**Pediatric** **cancer pts**	13	18	18	blood	NA	0	RF for BSI: severe neutropenia (<100/mm^3^; p=0.002) hospital-acquired infection (p<0.0001) breakthrough infection (p<0.0001)	NA	(22)	2017
**PICU**	0.3	NA	16	blood	NA	NA	NA	NA	(36)	2017
**Pediatric pts**	0.7		23	blood, respiratory, urine	35	NA	NA	NA	(37)	2017
**PICU**	5	31	91	blood, respiratory, soft tissues	61	16	RF for outcome: prior prolonged hospitalization (p=0.002) septic shock (p=0.003) mechanical ventilation (p=0.004) indwelling central vein catheter (p=0.03) prior use of steroids (p=0.04) prior use of carbapenems (p=0.004) mechanical ventilation (p=0.02)	outcome-related: combination of ciprofloxacin, TMP-SMX, and/or minocycline (p<0.001)	(38)	2018
**Critically ill children**	5	NA	68	blood	42	18	RF for outcome: prior prolonged hospitalization (p=0.03) nosocomial source of infection (p=0.02) septic shock (p<0.001) chemotherapy (p=0.007) carbapenems (p=0.05)	outcome-related: combination of ciprofloxacin, TMP-SMX, and minocycline (p<0.01)	(39)	2019
**Pediatric pts**	2	NA	104	blood, respiratory, soft tissues, CSF	NA	NA	NA	NA	(40)	2020
**Pediatric pts**	10	12	20	blood and/or catheter	33.3	NA	NA	NA	(41)	2020
**Pediatric pts**	7.3	128	161	blood, respiratory, CSF, wound	NA	3.9	RF for severe *S.maltophilia* infection: mechanical ventilation (p=0.021) prior ICU stay within 30 d (p=0.005) prior use of carbapenems (p=0.007)	NA	(42)	2020
**Pediatric pts**	2	NA	NA	blood	NA	NA	NA	NA	(43)	2020

ALC, absolute lymphocyte count; BAL, bronchio-alveolar lavage; BSI, blood stream infection; CSF, cerebral spinal fluid; d, days; ICU, intensive care unit; ID, infectious diseases; NA, not annotated; PICU, pediatric intensive care unit; pts, patients; TS, tracheostoma. Type of risk factor is underlined.

The exact route of acquisition of *S.maltophilia* often remains unknown. Nevertheless, isolation of the organism from mucosal surfaces of the respiratory and/or the lower gastrointestinal tract may herald later infection as many patients with *S.maltophilia* BSIs were reported to be colonized prior to infection (5, 17). Indeed, the oral microbiome has recently been described as a potential reservoir, and real-time monitoring of the oral *S.maltophilia* relative abundance has been suggested to identify patients at risk for invasive infection (30). In our limited cohort, concomitant colonization was detected in the majority of cases with invasive *S. maltophilia* infection, but overall, there was no apparent relationship between pharyngeal or respiratory colonization and invasive infection.

Similar to previous reports (20), the majority of invasive *S. maltophilia* infections in our cohort was associated with indwelling central venous catheters. Six of the ten patients were treated at the intensive care unit and four were on invasive ventilation. Intensive care, mechanical ventilation, and/or central venous catheterization have been identified as risk factors for *S. maltophilia* BSI and/or dismal outcome (**Table 2**). Several studies suggest a survival benefit for removal of indwelling central venous catheters (8, 9, 17, 47–49), and international guidelines strongly recommend prompt catheter removal in *S. maltophilia* associated BSIs (50), independent on whether the catheter is considered the source of the infection or being colonized secondary to ongoing bacteremia.

Patients with *S.maltophilia* BSIs often have polymicrobial infections (5), and their relative frequency in children seems to be higher as observed with *Pseudomonas aeruginosa* (35). In the cohort presented here, concomitant BSI occurred in 30% of patients with *S.maltophilia* infection, which is below the rate in previous series of pediatric patients (31, 33). Bacteria most commonly recovered in temporal context with *S.maltophilia* were coagulase-negative *Staphylococcus* and *Enterococcus* spp (8, 45). It remains unclear whether the detection of *S.maltophilia* is a consequence of appropriate antimicrobial therapy for other BSIs or whether the concurrent invasive infections simply reflect the sum of immunodeficiency in the affected patients.

In eight of the ten cases, *S.maltophilia* infection occurred as breakthrough infection in patients receiving broad-spectrum antibacterial agents. Prior use of carbapenems has been repeatedly described as a risk factor for *S.maltophilia* infection (12, 17, 44, 51–53), and cumulative carbapenem use has been identified to be associated with *S.maltophilia* in leukemia patients with altered oral microbiome (30). Similarly, in the majority of studies in pediatric patients investigating factors related with outcome, prior use of carbapenems was associated with dismal outcome of *S.maltophilia* BSI (22, 35, 39) (**Table 2**). As a consequence, clinicians should be aware that breakthrough infection with *S.maltophilia* may occur in severely ill patients being treated with carbapenems.

Antibacterial therapy for *S.maltophilia* infections is challenging because most clinical isolates are resistant to agents commonly used for empirical treatment of febrile neutropenia or documented infections by *Gram*-negative organisms, including extended-spectrum penicillins, third-generation cephalosporins, carbapenems, and aminoglycosides (53). In addition, current recommendations for treatment are based on historical evidence, case series, and *in vitro* susceptibility data rather than pharmacokinetic/pharmacodynamic considerations and results of controlled clinical trials (5). In general, TMP-SMX is the drug of choice for infections by susceptible *S.maltophilia* isolates based on its potent *in vitro* activity and documented clinical efficacy (10, 46). Nevertheless, susceptibility varies between geographic regions and resistance is an emerging threat (2, 5, 10, 16, 54, 55). Alternatives to treatment with TMP-SMX include fluoroquinolones and tigecycline (12, 13). However, in contrast to TMP-SMX, clinical breakpoints for these agents have not been defined, which makes a valid interpretation of *in vitro* susceptibility testing results with regards to the prediction of clinical efficacy difficult. In our study, seven of ten *S.maltophilia* initial isolates from patients with invasive infections were susceptible *in vitro* to TMP-SMX, and in one of these patients, isolates became resistant during treatment. Apart from primary or secondary resistance, further concerns in immunocompromised patients with cancer and/or allogeneic HCT include the myelotoxicity of therapeutic doses of TMP-SMX (18) and the widespread use of low and intermittent doses of the agent for antibacterial or anti-*Pneumocystis* prophylaxis that may result in the selection of resistant *S.maltophilia* strains (17). Indeed, based on emerging resistance, it has been suggested by individual experts to consider escalating therapies in immunocompromised or critically ill patients (49, 56). Previous observations on the use of fluoroquinolones against invasive *S.maltophilia* infections have demonstrated comparable patient survival relative to TMP-SMX (12, 13, 57), and quinolone prophylaxis in adult cancer patients has been associated with a reduced incidence of invasive *S.maltophilia* infections (58). Nevertheless, quinolone monotherapy for *S.maltophilia* BSIs should be critically reflected (19), as rapid emergence of resistance to these agents has been observed both *in vitro* and *in vivo* (5, 59).

Considering the small number of patients, the 30-day mortality rate of 30% in patients with invasive *S.maltophilia* infections in our study is in line with 30-day mortality rates of *S.maltophilia* BSIs of 33% and 38% reported by others (17, 60). In pediatric studies, the reported all-cause mortality rates in patients with *S.maltophilia* infections range from 12.5% to 61% with an attributable mortality of 0% to 18%, respectively (22, 31, 33, 37–39, 41) (**Table 2**). Many studies across all age groups have reported risk factors for mortality associated with *S.maltophilia* BSIs including prolonged hospitalization prior to BSI onset, previous exposure to antimicrobial agents, use of indwelling medical devices, a compromised health status, complex medical care, granulocytopenia and/or transplantation, and inappropriate therapy (5, 8–10, 15, 17, 18, 36, 48, 61–64). While the limited number of patients included precluded robust statistical assessments, the presence of pneumonia and admission to the intensive care unit were both significantly associated in explorative analyses with mortality in the present study (**Supplementary Table 1**). Nevertheless, as it is often difficult to distinguish between colonization and infection, identification of risk factors for mortality is ultimately limited to BSIs and may not consider the full spectrum of diseases caused by the organism (7).

To conclude, as reflected in this limited series of heterogenous patients, defined therapeutic strategies for invasive *S.maltophilia* infections in immunocompromised pediatric patients, including those with cancer and/or allogeneic HCT, so far lack uniformity but remain an important goal. Clinicians should be aware that breakthrough infections by S.*maltophilia* may occur during the administration of broad-spectrum antibiotics, especially following carbapenem use, and that these infections may be associated with fulminant and fatal pulmonary hemorrhage, in particular in allogeneic HCT patients (29). Detection of BSI by *S.maltophilia* should prompt the removal of indwelling central venous catheters and the immediate initiation of therapeutic doses of TMP-SMX. Initial combination with second generation fluoroquinolones and tigecycline until return of resistance testing and achievement of a stable clinical response may be considered in view of the high case fatality rates.

## Data Availability Statement

The data analyzed in this study is subject to the following licenses/restrictions: Clinical data. Requests to access these datasets should be directed to (andreas.groll@ukmuenster.de).

## Ethics Statement

Written informed consent was obtained from the individual(s), and minor(s)’ legal guardian/next of kin, for the publication of any potentially identifiable images or data included in this article.

## Author Contributions

Retrospective analysis of single-center data and literature review was conducted by SKZ, supported by HH and with clinical input by CR, AH, KM, AR, and AHG. Identification and susceptibility testing by NJF, whole genome sequencing based typing was performed and analyzed by SK. Statistical analysis was performed by AR, SKZ and AHG. Manuscript was written by SKZ and was edited by SK and AHG. All authors contributed to the article and approved the submitted version.

## Funding

The study was funded by internal resources.

## Conflict of Interest

The authors declare that the research was conducted in the absence of any commercial or financial relationships that could be construed as a potential conflict of interest.

## Publisher’s Note

All claims expressed in this article are solely those of the authors and do not necessarily represent those of their affiliated organizations, or those of the publisher, the editors and the reviewers. Any product that may be evaluated in this article, or claim that may be made by its manufacturer, is not guaranteed or endorsed by the publisher.

## Supplementary Material

Supplementary Table 1 Explorative statistics of the association between demographic or clinical factors with non-survival.
