# Supplementary material for: Stenotrophomonas maltophilia Infections in Pediatric Patients – Experience at a European Center for Pediatric Hematology and Oncology
Source: Front Oncol. 2021 Oct 12;11:752037. doi: 10.3389/fonc.2021.752037 (PMC8547273; doi:10.3389/fonc.2021.752037)
Supplement: Supplementary Table 1 — Explorative statistics of the association between demographic or clinical factors with non-survival. [file Table_1.docx]

**Supplemental Table 1.** Explorative statistics of the association between demographic or clinical factors with non-survival of ten pediatric patients with oncological or hematological disease including patients with autologous or allogeneic hematopoietic cell transplantation and invasive *S.maltophilia* infections.

|  | Parameter | Invasive infections (*n*) | Total events (*n*) | Comparison of parameters  by Fisher’s Exact Test |
| --- | --- | --- | --- | --- |
| Patient characteristics | **Sex**  Male  Female | **10**  5  5 | 1  4 | 0.206 |
|  | **Age** (years)  0-9.4  >9.4 | **10**  4  6 | 1  4 | 0.523 |
|  | **Allogeneic HCT**  Yes  No | **10**  5  5 | 3  2 | 1.000 |
| Clinical aspects | **Pneumonia**  Yes  No | **10**  4  6 | 4  1 | **0.047** |
|  | **ICU support**  Yes  No | **10**  6  4 | 5  0 | **0.047** |
|  | **Mechanical ventilation**  Yes  No | **10**  4  6 | 3  2 | 0.523 |
|  | **TMP-SMX**  Yes  No | **10**  2  8 | 1  4 | 1.000 |
| Antibiotic treatment | **Quinolones**  Yes  No | **10**  7  3 | 2  3 | 0.166 |
|  | **Tigecycline**  Yes  No | **10**  5  5 | 2  3 | 1.000 |
|  | **Colistin**  Yes  No | **10**  3  7 | 1  4 | 1.000 |
|  | ***S.maltophilia* colonization**  Yes  No | **10**  7  3 | 3  2 | 1.000 |
|  | **Concomitant BSI**  Yes  No | **10**  3  7 | 2  3 | 1.000 |
| Blood parameters | **Absolute neutrophile count** (10^3^/µL)  0-0.5  >0.5 | **10**  7  3 | 4  1 | 1.000 |
|  | **Absolute neutrophile count** (10^3^/µL)  0-0.5  >0.5 | **10**  7  3 | 4  1 | 1.000 |
| BSI, blood stream infection; HCT, hematopoetic stem cell transplantation; ICU, intensive care unit; TMP-SMX, trimethoprim-sulfamethoxazole | | | | |
